# An Observational Study to Evaluate Readability and Reliability of AI-Generated Brochures for Emergency Medical Conditions

**DOI:** 10.7759/cureus.68307

**Published:** 2024-08-31

**Authors:** Adithya S, Shreyas Aggarwal, Janani Sridhar, Kavya VS, Victoria P John, Chaihthanya Singh

**Affiliations:** 1 Medical School, Ramaiah Medical College, Bangalore, IND; 2 Geriatrics, Prince Charles Hospital, Cwm Taf Morgannwg University Health Board, Merthyr Tydfil, GBR; 3 Preventive Care, Apollo Hospitals, Chennai, IND; 4 General Medicine, Sree Uthradom Thirunal Academy of Medical Sciences, Trivandrum, IND; 5 Medical Services, Kauvery Hospital, Chennai, IND; 6 Anatomy, Dr. D.Y. Patil Medical College and Hospital, Pune, IND

**Keywords:** readability measures, google gemini, chatgpt, discern score, flesch-kincaid, syncope, life threatening anaphylaxis, heart attack, #patient education, ai-generated brochures

## Abstract

Introduction

The study assesses the readability of AI-generated brochures for common emergency medical conditions like heart attack, anaphylaxis, and syncope. Thus, the study aims to compare the AI-generated responses for patient information guides of common emergency medical conditions using ChatGPT and Google Gemini.

Methodology

Brochures for each condition were created by both AI tools. Readability was assessed using the Flesch-Kincaid Calculator, evaluating word count, sentence count and ease of understanding. Reliability was measured using the Modified DISCERN Score. The similarity between AI outputs was determined using Quillbot. Statistical analysis was performed with R (v4.3.2).

Results

ChatGPT and Gemini produced brochures with no statistically significant differences in word count (p= 0.2119), sentence count (p=0.1276), readability (p=0.3796), or reliability (p=0.7407). However, ChatGPT provided more detailed content with 32.4% more words (582.80 vs. 440.20) and 51.6% more sentences (67.00 vs. 44.20). In addition, Gemini's brochures were slightly easier to read with a higher ease score (50.62 vs. 41.88). Reliability varied by topic with ChatGPT scoring higher for Heart Attack (4 vs. 3) and Choking (3 vs. 2), while Google Gemini scored higher for Anaphylaxis (4 vs. 3) and Drowning (4 vs. 3), highlighting the need for topic-specific evaluation.

Conclusions

Although AI-generated brochures from ChatGPT and Gemini are comparable in readability and reliability for patient information on emergency medical conditions, this study highlights that there is no statistically significant difference in the responses generated by the two AI tools.

## Introduction

Artificial Intelligence (AI) has been a huge part of our lives since its birth. Nowadays, almost each and every person resorts to searching the net for their symptoms once they fall sick and uses the treatment option suggested by the AI chatbot. This has proven to be beneficial in some, but also disastrous for many. The advent of AI has made a huge difference in the medical world, although it can only be used as a tool of assistance rather than an actual consultant giving medical advice.

Myocardial infarction (MI) is a term used for an event of heart attack which is due to the formation of plaques in the interior walls of the arteries resulting in reduced blood flow to the heart and injuring heart muscles because of lack of oxygen supply [1]. Patient education is defined as "the process by which health professionals and others impart information to patients that will alter their health behaviors or improve their health status" [2]. Patient education for heart attack empowers individuals by raising awareness of risk factors, symptoms, and treatment options. It promotes adherence to medications and lifestyle changes crucial for recovery and prevention. Education also supports psychosocial well-being and involves caregivers, fostering a proactive approach to heart health management [3].

Choking is a serious and potentially life-threatening condition that can occur when an object, usually food, becomes lodged in the throat or windpipe, blocking airflow. It can lead to death if the object is not removed quickly [4]. A patient education guide on choking is crucial for prevention, recognizing symptoms, providing immediate first aid, and ensuring post-incident care. It empowers patients and caregivers, builds confidence, and reduces anxiety by outlining safe eating practices, emergency response techniques like the Heimlich maneuver, and the importance of medical follow-up [5].

Anaphylaxis is a severe allergic reaction requiring immediate treatment with epinephrine and emergency medical attention. Symptoms include skin reactions, respiratory distress, and cardiovascular issues. Prevention involves avoiding known allergens, carrying an epinephrine auto-injector, and educating others on emergency response. It is an acute, potentially life-threatening systemic hypersensitivity reaction which is classically mediated by IgE [6]. Patient education on anaphylaxis is vital for recognizing symptoms, using epinephrine correctly, avoiding triggers, and following an action plan. It reduces anxiety, ensures follow-up care, and prevents recurrence. Methods include printed materials, digital resources, and interactive sessions. Effective education improves management and reduces severe reactions.

Drowning is the process of experiencing respiratory impairment from submersion or immersion in liquid. It is one of the few common emergency situations we may come across and it has been estimated that > 90% of drownings are preventable [7]. Patient education on drowning prevention is vital for recognizing risks, ensuring supervision, using safety equipment, and learning to swim. It guides emergency response, including calling for help and performing CPR. Education reduces incidents, empowers individuals, and encourages community involvement in water safety programs and resources like CPR courses [8].

Syncope is a temporary loss of consciousness due to transient global cerebral hypoperfusion [9]. Patient education on syncope includes recognizing symptoms, maintaining hydration, managing posture, and avoiding triggers. Key actions during an episode are lying down and elevating legs. Long-term management involves lifestyle changes, medications, and regular medical check-ups. Accessing educational resources and support groups can further aid in managing syncope [10]. Patient education on syncope is vital for understanding its causes, recognizing symptoms, and implementing preventive measures. It guides immediate response actions, encourages medical evaluation, and promotes lifestyle adjustments. Education improves safety, quality of life, and empowers patients with the knowledge and tools for effective management.

Artificial Intelligence (AI) is transforming emergency healthcare response systems by leveraging machine learning algorithms to analyze extensive data sets, such as patient records, vital signs, and historical trends. These algorithms can detect patterns, predict outcomes, and optimize resource allocation, enhancing accuracy and reliability. Although complex, these AI models are not meant to replace human expertise but to complement it. By integrating AI into clinical practice, healthcare professionals and patients can benefit from improved efficiency and access to information, leading to better-informed decisions and enhanced patient outcomes through timely and accurate responses.

## Materials and methods

This study was a cross-sectional study, which took place over one month, from June to July 2024, including designing the study, collecting data, and manuscript writing. The study only utilized data generated by ChatGPT and Google Gemini, and did not involve human participants, thus exempting the study from obtaining ethical approval.

To collect data, five acute medical emergency conditions - choking, heart attack, anaphylactic shock, drowning and syncope - were chosen. Two AI tools, ChatGPT (version 4.0, March 2024) and Google Gemini (version 1.2, May 2024), were tasked with creating brochures for these conditions. The specific prompts given were: *"Write a patient education guide for choking", "Write a patient education guide for drowning”, "Write a patient education guide for heart attack”, “Write a patient education guide for syncope", and "Write a patient education guide for anaphylactic shock.” *The AI-generated responses were compiled into a Microsoft Word document.

These responses were then assessed using several criteria. The Flesch-Kincaid Calculator was utilised to measure word count, sentence count, readability, and ease of understanding [11]. Additionally, the similarity of the content was evaluated with the Quillbot Plagiarism Tool [12]. The reliability of the scientific information provided was assessed using the Modified DISCERN score, which evaluates the quality of written health information, particularly regarding treatment choices. In the modified DISCERN scale, each question was scored either 0 or 1 point. A total score of 5 indicates high reliability whereas a 0-point demonstrates low reliability in the scoring system [13].

For the statistical analysis, the data were transferred to a Microsoft Excel sheet and analysed using R version 4.3.2 (R Core Team, 2023). An unpaired T-test was conducted to compare the responses from ChatGPT and Google Gemini, with a p-value of less than 0.05 indicating significance. Pearson’s Coefficient of Correlation was used to assess the relationship between the readability scores and the reliability scores of the information provided.

## Results

ChatGPT and Google Gemini were employed to generate brochures on patient education for five critical medical emergencies: Heart Attack, Choking, Anaphylaxis, Drowning, and Syncope. The analysis aimed to compare the generated content across several characteristics to determine if there were significant differences in the quality and readability of the materials produced by these AI tools. This study provided a comprehensive comparison of the two AI tools in terms of word count, sentence count, readability, and reliability of the information presented.

Table 1 shows the characteristics of the responses generated by ChatGPT and Google Gemini. It was observed that the brochures generated by ChatGPT had a higher mean word count (n=582.80) and a greater number of sentences on average (n=67) compared to those generated by Google Gemini [n(words)=440.20, n(sentences)=20.66].

**Table 1:** Characteristics of responses generated by ChatGPT and Google Gemini *t-test. P-values <0.05 are considered statistically significant.

Variables	ChatGPT		Google Gemini		P value*
	Mean	Standard Deviation	Mean	Standard Deviation	
Words	582.80	136.96	440.20	193.82	0.2199
Sentences	67.00	21.74	44.20	20.66	0.1276
Average Words per Sentence	9.14	2.76	10.56	2.20	0.3961
Average Syllables per Word	1.84	0.20	1.72	0.15	0.3074
Grade Level	9.12	2.77	8.84	1.92	0.8575
Ease Score	41.88	16.64	50.62	12.71	0.3796
Similarity %	34.20	27.33	13.42	10.21	0.1711
Reliability Score	2.80	0.84	3.00	1.00	0.7407

The analysis showed no statistically significant difference in most of the domains studied. Specifically, there was no significant difference in the word count (p = 0.2199), sentence count (p = 0.1276), average words per sentence (p = 0.3961), average syllables per word (p = 0.3074), grade level (p = 0.8575), ease score (p = 0.3796), similarity percentage (p = 0.1711), and reliability score (p = 0.7407).

**Figure 1:**
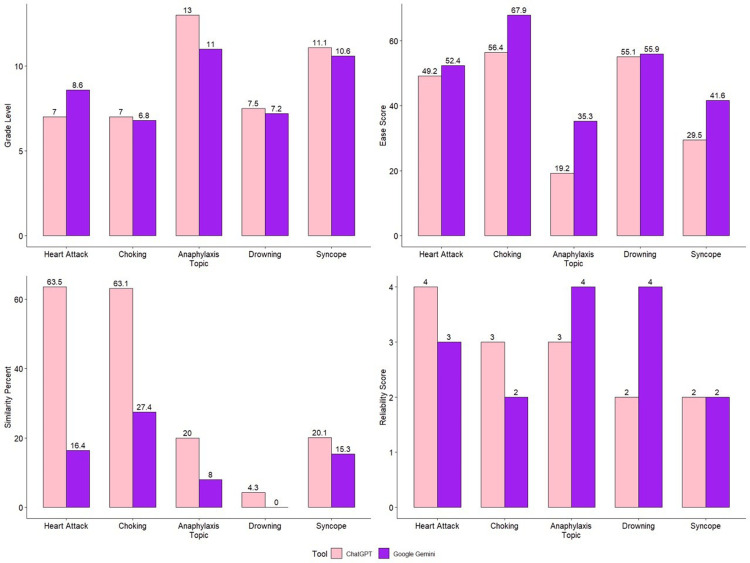
Graphical representation of comparison between grade level, ease score, similarity percent and reliability score, for the patient education guide generated by ChatGPT and Google Gemini.

Figure 1 provides a graphical representation of the comparison between grade level, ease score, similarity percentage, and reliability score for the patient education guides generated by ChatGPT and Google Gemini.

The grade level comparison shows that the responses were generally similar, with one notable discrepancy in the Anaphylaxis topic, where ChatGPT's content was rated at a higher grade (13) level compared to Google Gemini's (11), indicating that ChatGPT's information might be slightly more complex for patients to understand for this particular topic.

When comparing the ease score, ChatGPT's brochures scored slightly lower across all topics than those from Google Gemini across all topics, with a pronounced difference in the Choking topic (EaseChatGPT=66.4, EaseGoogle=67.9), indicating that Google Gemini's content was easier to read.

The reliability score did not exhibit a consistent pattern across topics. ChatGPT demonstrated higher reliability for Heart Attack (4) and Choking (3), while Google Gemini's brochures scored better for Anaphylaxis (4) and Drowning (4). The Syncope topic showed identical reliability scores (2) for both AI tools.

On comparing the similarity percentage between the educational guides generated, it was noted that ChatGPT had a significantly higher percentage of similarity across all topics compared to Google Gemini.

## Discussion

A cross-sectional study conducted to compare responses generated by two AI tools ChatGPT and Google Gemini for brochures on patient education for heart attack, choking, anaphylaxis, drowning and syncope revealed that there is no statistically significant difference between the two AI tools. The grade level used by ChatGPT was 9.12 and the grade level used by Google Gemini was found to be 8.84 (p value=0.8575). The ease score for ChatGPT was 41.88 and 50.62 for Google Gemini (p-value=0.3796). Similarity % was found to be 34.20 for ChatGPT and 13.42 for Google Gemini (p-value=0.1711). Finally, the reliability score was 2.80 for ChatGPT compared to 3.00 for Google Gemini (p-value=0.7407). From the above-mentioned statistics, both ChatGPT and Google Gemini can be used easily to get a basic gist of the patient education guide for emergency conditions like heart attack, choking, anaphylaxis, drowning and syncope.

AI has the potential to bring about positive changes in healthcare and empower patients by providing them with more control over their health. In recent years, AI has been used to improve the delivery of healthcare in a variety of ways, from providing personalized health information to enabling virtual consultations and remote monitoring [14].

Many medical articles on the net are hard to read and interpret for a common man, AI helps by summarizing the information available on the net and making it easy to read. Our study was focused on assessing the ease of reading the brochures generated by ChatGPT and Google Gemini. The difficulty level of the information was assessed by the Flesch reading-ease score (FRES) test. For an article to be read by a high school student the Flesch score should be at least 50-60 [15].

The present study found the ease score to be 41.88 for ChatGPT and 50.62 for Google Gemini which denotes that both AI articles could be read by a high schooler, although the ChatGPT article could be a little difficult to comprehend by a 10th grader. One study done by Gordon et al. found the ChatGPT’s Flesch-Kincaid grade level to be 13.6, which did not reach the eighth grade readability recommended for patient-facing materials [16].

As the chatbots are trained using pre-existing literature available on the net, they can generate similar sentences leading to plagiarism. Plagiarism in medical practice is a serious ethical violation that can have significant consequences. It can damage the reputation of healthcare professionals and institutions, leading to a loss of trust. Medical professionals may face disciplinary actions from licensing boards, including suspension or revocation of licenses. Involvement in plagiarism can result in loss of research funding or grants. Misrepresentation of research or clinical practices can negatively affect patient outcomes and public health.

From our study the similarity % was found to be higher for ChatGPT compared to Google Gemini. A study by Sallam revealed concerns regarding ChatGPT use which were stated in 58/60 (96.7%) records including ethical, copyright, transparency, and legal issues, the risk of bias, plagiarism, lack of originality, inaccurate content with risk of hallucination, limited knowledge, incorrect citations, cyber security issues, and risk of infodemics [17].

The Modified DISCERN Score is a tool used to evaluate the quality of health information, particularly online resources. It is scored from 1 to 5, with higher scores indicating better quality. Our study found the average DISCERN scores for ChatGPT and Google Gemini to be 2.80 and 3 respectively, which signifies that the quality was moderate and can be improved.

This finding can be compared to a study by Durmaz Engin et al., which revealed that ChatGPT had the highest DISCERN score along with Flesch-Kincaid grade level, amongst other AI models like Bing AI and Google Gemini in educating families about retinopathy of prematurity [18].

Limitations

Only two AI tools were used in this study, other AI tools could have been assessed to get better results. More diseases could have been included for better clarity. The chatbots are updated very frequently and this may result in using the older AI version. In a few cases the AI may not provide the new updated content in medical science, as it can be hard to access.

## Conclusions

This study highlights that there is no statistically significant difference in the ease score, grade level, similarity percent and reliability score of responses generated by ChatGPT and Google Gemini for patient information brochures on common emergency medical conditions.

Seeing that this study covers only two of the AI tools, there is a scope for further study using other AI tools. Moreover, there is a need to update the AI tools in order to provide information based on the latest and verified guidelines.
